# Arms race of physical defences: hooked trichomes of *Macaranga* ant-plants kill lycaenid caterpillars, but one specialist has a counter-defence

**DOI:** 10.1098/rsbl.2025.0005

**Published:** 2025-06-06

**Authors:** Ritabrata Chowdhury, T. Ulmar Grafe, Faizah Metali, Walter Federle

**Affiliations:** ^1^ Department of Zoology, University of Cambridge, Cambridge, Cambridgeshire, UK; ^2^ Faculty of Science, Universiti Brunei Darussalam, Bandar Seri Begawan, Brunei-Muara District, Brunei Darussalam

**Keywords:** physical defence, counter-adaptation, *Macaranga* ant-plants, trichomes, lycaenids, arms race

## Abstract

The coevolution of insects and chemical plant defences has been described as an arms race, but it is unclear whether physical plant defences can produce similar outcomes. Here, we report a previously unknown interaction from the mutualism between ants and *Macaranga* trees. Although *Macaranga* trees are well protected against herbivory by aggressive ants, caterpillars of the genus *Arhopala* (Lepidoptera: Lycaenidae) can feed on the leaves by appeasing the ants with nectar-like secretions. One ant-plant species, *M. trachyphylla*, bears hooked trichomes on its green surfaces. When placed on *M. trachyphylla* stems or petioles, *Arhopala* caterpillars associated with other *Macaranga* species (*A. major, A. dajagaka* and *A. zylda*) were quickly arrested by the sharp trichomes that pierced their cuticle, resulting in death by rapid blood loss and removal by ants. In striking contrast, *A. amphimuta* caterpillars, which occur naturally on *M. trachyphylla*, could easily walk over the hooked trichomes without any injury. As hooked trichomes are a novel trait within *Macaranga*, this interaction provides an example of de novo evolution of a physical plant defence, which in turn has been overcome by a specialist herbivore. Our study suggests that physical plant defences can lead to evolutionary arms races similar to those for chemical defences.

## 1. Introduction

Plants and insects have coexisted for more than 400 million years, and over this period, have developed complex interactions that play a critical role in terrestrial ecosystems [1–3]. While some of these relationships are mutually beneficial, such as pollination or seed dispersal, the majority involve insects feeding on plants, and plants defending themselves against herbivory [3]. The mutual adaptation of chemical plant defences and insect herbivores has been described as an evolutionary arms race that has led to the diversification of both insects and plants [2,4]. Do such arms races also occur for physical defences?

One of the physical defences of plants against herbivores are trichomes, hair-like appendages produced by the epidermis. Trichomes can protect against herbivory by limiting insect access to leaf or stem tissues [5–7], reducing insect attachment [8], exposing insects to toxic or sticky substances [9,10] or puncturing the insect’s cuticle [11,12]. Trichomes can also decrease the rate of tissue ingestion and thereby the herbivore’s growth [6,7] and prevent insect oviposition or reduce the adhesion of eggs to the plant surface [13,14]. Sharp hooked trichomes are considered one of the most effective forms of physical defence in plants. In *Passiflora* vines, they can injure or kill caterpillars by piercing their cuticle [11,15]. Similarly, hooked trichomes on the leaves of common bean (*Phaseolus vulgaris*) can injure insects in different parts of their bodies, leading to their death by haemolymph loss or starvation [12,16–18].

Some insect herbivores have in turn evolved adaptations to deal with trichomes. These adaptations can be morphological, such as elongated legs, to avoid contact with the sticky heads of glandular trichomes [19] or tarsal hooks for anchoring to trichomes [20,21]. Insects can also adapt behaviourally by walking ‘on tiptoe’ between trichomes [19], removing dense trichome covers before feeding on leaves [22], or spinning silk mats to move on trichome-bearing surfaces without being punctured [23].

Here, we report an interaction involving trichomes as a physical defence in ant-associated trees of the palaeotropical genus *Macaranga* (Euphorbiaceae). Most *Macaranga* species are myrmecophilic and protect themselves against herbivory by attracting non-specific foraging ants, but *ca* 30 species in Southeast Asia are myrmecophytic and permanently house specialized ant partners nesting in their hollow stems. Although the ants efficiently keep away most herbivores, several species of *Arhopala* caterpillars (Lepidoptera: Lycaenidae) have outwitted the ants’ defences by providing them with nectar-like secretions from their myrmecophilous organs and feeding on *Macaranga* leaves [24,25]. One *Macaranga* ant-plant species in Borneo, *M. trachyphylla*, has stems and petioles densely covered by hooked trichomes, giving the surfaces a sandpaper-like feel. To our knowledge, *M. trachyphylla* is the only one of more than 300 described *Macaranga* species for which this trait is known [26]. The functional significance of these trichomes is still unknown. We therefore investigated the effect of *M. trachyphylla* trichomes on *Arhopala* caterpillars.

## 2. Results

**Figure 1.**
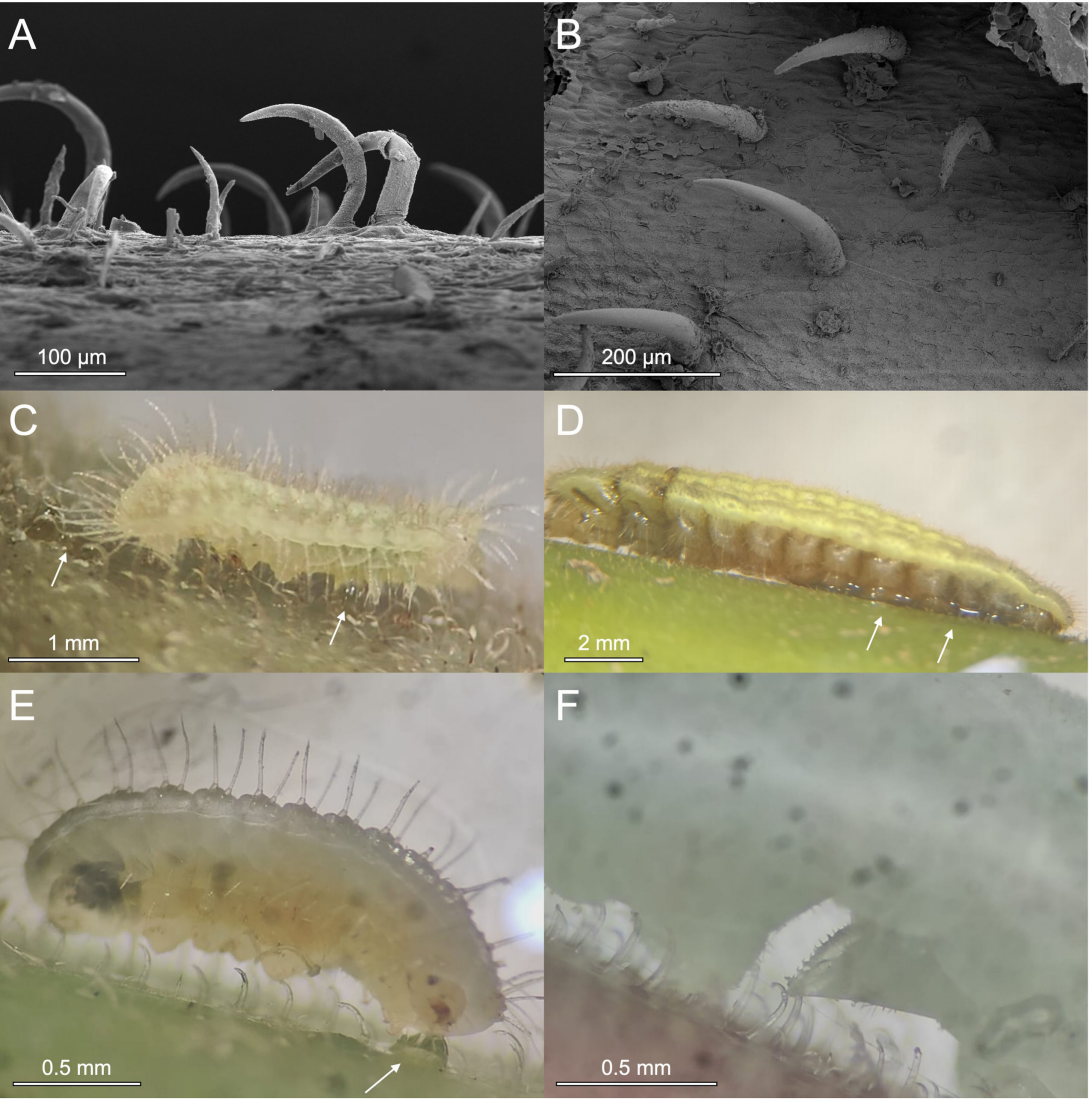
(A,B) Scanning electron micrographs of hooked trichomes on the petiole (A) and stem (B) of *M. trachyphylla*; (C) *Arhopala major* caterpillar (second instar) injured by *M. trachyphylla* trichomes, with haemolymph oozing from the wounds (arrows); (D) same as (C), but fourth instar; (E) first to second instar *A. dajagaka* caterpillar, with haemolymph oozing from the wounds (arrow); and (F) third instar *A. zylda* caterpillar, showing the proleg footpad pierced by a hooked trichome.

**Figure 2.**
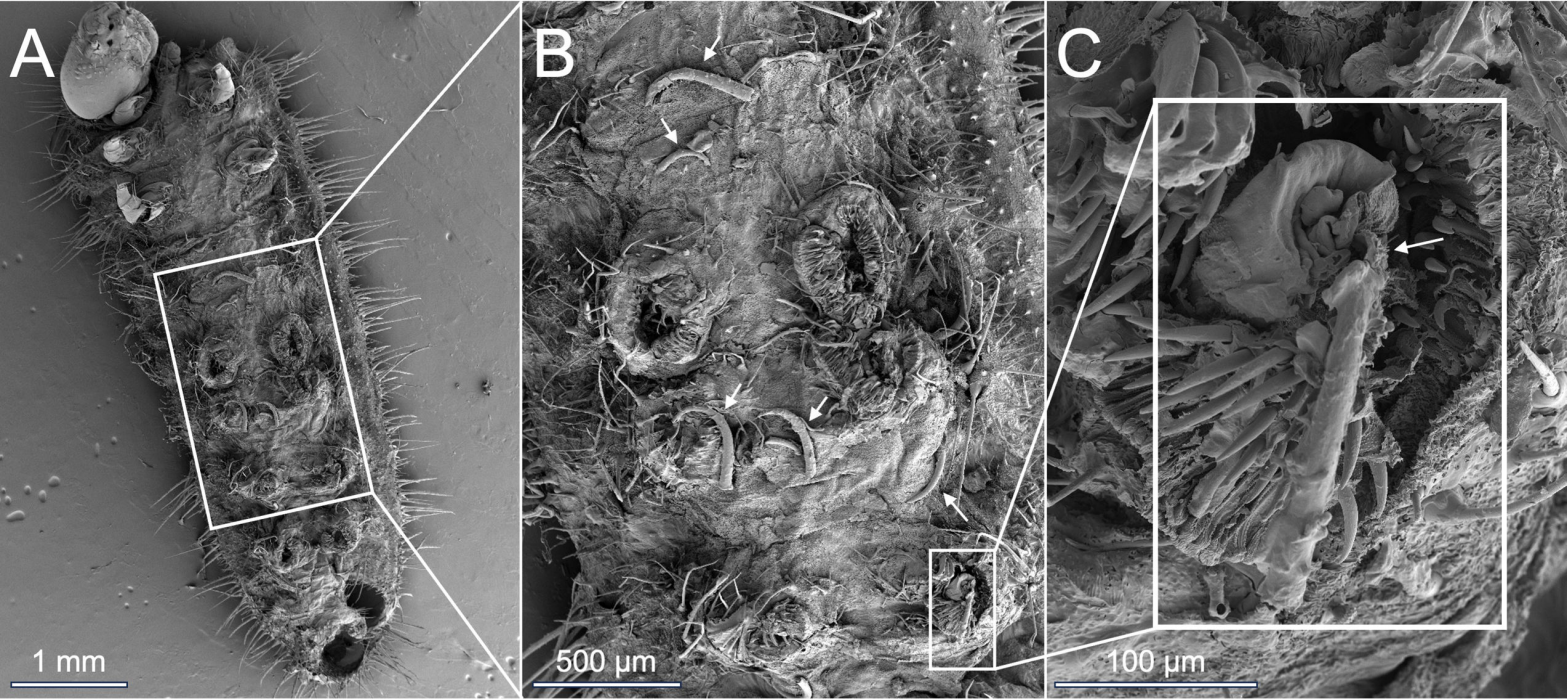
(A–C) Scanning electron micrographs of the ventral side of a third to fourth instar *A. major* caterpillar after walking on the trichome-bearing stem surface of *M. trachyphylla*; (B,C) magnified images showing several broken trichomes (arrows in B) on the surface of the cuticle, and a hooked trichome piercing the cuticle of the third left proleg footpad (arrow in C).

**Figure 3.**
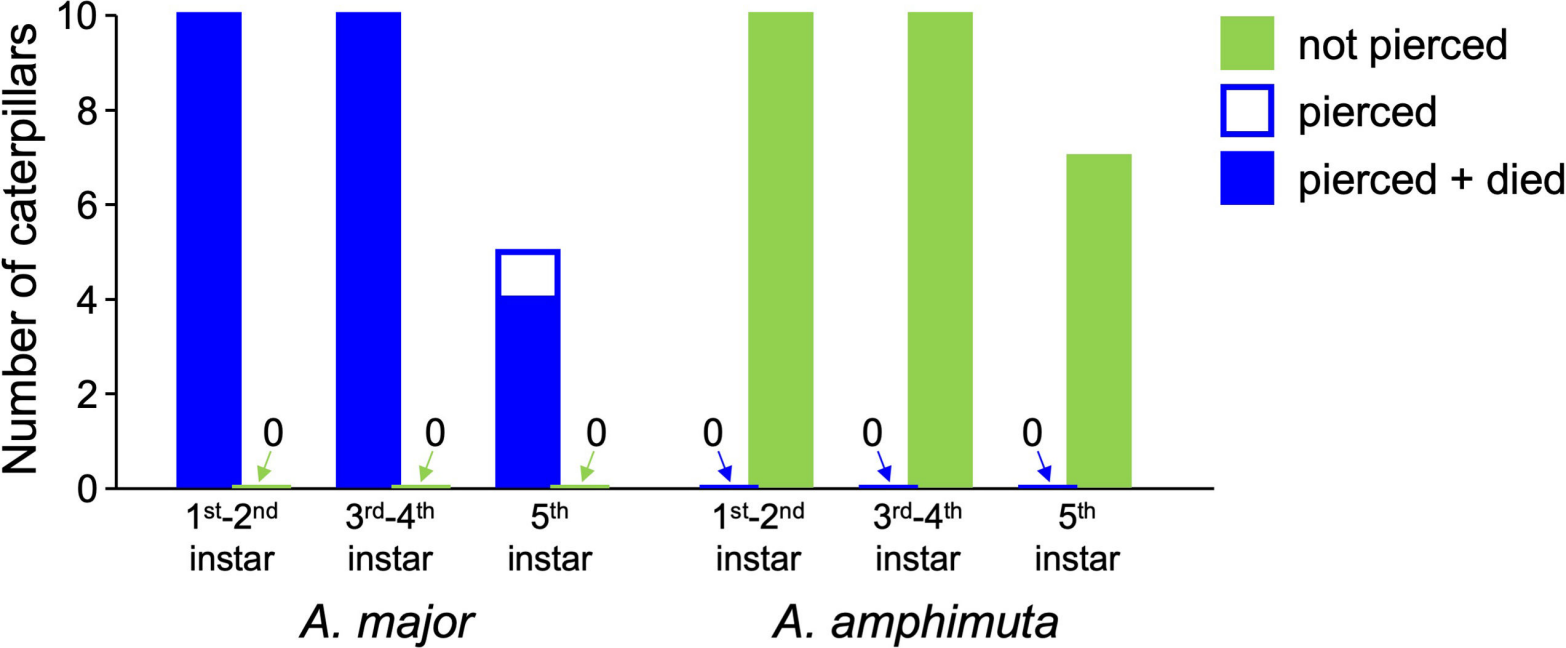
Effect of hooked trichomes on petioles and stems of *M. trachyphylla* on *A. major* and *A. amphimuta* caterpillars.

*Arhopala major* caterpillars attempting to walk on petioles or stems of *M. trachyphylla* were rapidly arrested by the sharp hooked trichomes (figure 1A,B). As soon as they became stuck on a trichome, the caterpillars could only move about 1−2 mm further. The sharp trichome tips pierced the caterpillars’ prolegs and/or abdomen and thorax (figures 1C,D and 2B,C; electronic supplementary material, videos S1, S2), leading to the oozing out and rapid loss of haemolymph. All *A. major* caterpillars of the first to fourth instar (*n* = 20) died very quickly, with death usually occurring within half an hour of the injury (figure 3). The only *A. major* caterpillar not immediately killed on *M. trachyphylla* stems was from the final (fifth) instar (one out of five caterpillars; figure 3); it was also arrested by the trichomes, but due to its larger body size, only the prolegs were pierced, so it did not die as a result of the interaction.

We found very similar effects in *A. zylda* and *A. dajagaka* caterpillars, although fewer specimens were available from these species (figure 1E,F). All the caterpillars were arrested by the trichomes; *A. dajagaka* died due to loss of haemolymph, whereas the *A. zylda* caterpillars (electronic supplementary material, video S3) could not move more than 1−2 mm but did not die as they were only injured in their prolegs.

In striking contrast, *A. amphimuta* caterpillars of all instars were able to move easily over the hooked trichomes on *M. trachyphylla* stems and petioles, and no injuries were observed in any of the caterpillars (figure 3; electronic supplementary material, video S4). We observed that the prolegs or the body wall often caught and pulled on the hooked trichomes, but the trichomes did not puncture the cuticle. The caterpillars sometimes raised their prolegs high to free them from the trichomes, but we also observed this behaviour in the other *Arhopala* species tested. *Arhopala amphimuta* caterpillars were always able to free their prolegs from the trichomes without being pierced. In contrast to *M. trachyphylla* stems and petioles, the leaves of *M. trachyphylla* were harmless for all the *Arhopala* species; all caterpillars could walk on them without being injured. This is likely due to the significantly lower density of hooked trichomes on the leaves compared to stems and petioles (stems: 46.5 ± 17.6 mm^-2^, petioles: 60.5 ± 26.6 mm^−2^, leaf underside: 8.7 ± 4.9 mm^−2^, leaf upper side: 3.4 ± 0.7 mm^−2^, *n* = 5 samples each; see electronic supplementary material, figures S5–S8).

We tested whether *A. major* caterpillars would be able to cope with the mutualistic *Crematogaster* plant-ants living on *M. trachyphylla* trees. When placed on the youngest leaves, the caterpillars were mostly ignored or attended by the ants (52% and 34% of caterpillar–ant interactions, respectively); they were only occasionally attacked (14% of interactions). However, when placed on the stem, all *A. major* caterpillars (*n* = 10) became stuck in the trichomes within only 5 min, were punctured by them and started to lose haemolymph. Once the caterpillars were injured, the ants’ behaviour changed; there were quickly many ants that attacked and removed the caterpillar (*n* = 10), probably due to the injury and because it was no longer able to use its myrmecophilous organs.

## 3. Discussion

Our results show that the hooked trichomes of *M. trachyphylla* act as a powerful physical defence against *A. major*, *A. zylda* and *A. dajagaka* caterpillars, and that the *Arhopala* species naturally occurring on *M. trachyphylla*, *A. amphimuta*, is resistant to them. The sharp trichomes pierce the caterpillars’ cuticle, leading to rapid blood loss and death mostly occurring within minutes.

The action of the hooked trichomes of *M. trachyphylla* against *Arhopala* caterpillars and the presence of a species that is immune to this physical defence represent a striking evolutionary convergence with the interaction of *Passiflora* trichomes and *Heliconius* caterpillars discovered by Gilbert [11]. *Heliconius* caterpillars are also stopped, punctured and killed by the sharp hooked trichomes on *Passiflora* leaves, and there is one species, *H. charithonia*, that is resistant and capable of feeding on trichome-bearing plants [15,27]. However, the *Arhopala–M. trachyphylla* interaction discovered here differs in some important ways from the *Passiflora–Heliconius* system.

Firstly, *M. trachyphylla* trees are ant-plants permanently inhabited by colonies of *Crematogaster* ants. These ants provide a powerful biotic defence that the herbivorous *Arhopala* caterpillars have to cope with. The five closely related *Arhopala* species associated with *Macaranga* (which form the *amphimuta amphimuta* subgroup [28]), each occur on a different set of *Macaranga* species (table 1). It is still unclear whether this host specificity is based on ant defences or on chemical or physical plant traits. We found that *A. major* caterpillars were not strongly rejected by the plant-ants living on *M. trachyphylla*, and similar observations have been reported for *A. zylda* and *A. dajagaka* [31]. It is also unlikely that the specificity of *A. major* for *M. gigantea* and absence on *M. trachyphylla* is due to defensive plant chemicals, because *A. major* caterpillars can feed on *M. trachyphylla* leaves and successfully pupate [30]. Our findings show that the absence of *A. major, A. zylda* and *A. dajagaka* caterpillars on *M. trachyphylla* can be fully explained by the sharp, hooked trichomes on the stems and petioles.

**Table 1.** *Macaranga* host plant species of the studied *Arhopala* species.

*Arhopala* species	recorded *Macaranga* host plant species	references
*A. amphimuta*	*M. trachyphylla, M. bancana* *M. hullettii, M. havilandii*	[24,29,30]
*A. zylda*	*M. beccariana, M. hypoleuca*	[24,29]
*A. dajagaka*	*M. rufescens*	[29]
*A. major*	*M. gigantea*	[29]
*A. moolaiana*	*M. hullettii*	[24]

A second difference to *Passiflora* is that the trichome defence of *M. trachyphylla* is restricted to the petioles and stems. *Arhopala major* caterpillars can feed on *M. trachyphylla* leaves in the laboratory [30], but under natural conditions, the trichomes on petioles and stems are sufficient to prevent them from completing their development. Assuming an *A. major* egg is laid on a young leaf of *M. trachyphylla*, the emerging young caterpillar will at some point have to move to the next leaf via the petiole and stem. In this process, the caterpillar will be trapped and killed by the trichomes. This indicates that while the hooked trichomes of *Passiflora* have both pre- and post-ingestive effects on *Heliconius* caterpillars [32], the effects are purely pre-ingestive and external in the *Arhopala/Macaranga* interaction.

It has long been proposed that there is a coevolutionary arms race between the chemical defences of plants and insect counter-adaptations [2,4,33]. Until now, it is unclear whether physical defences can trigger similar arms races. The interaction of hooked trichomes in *M. trachyphylla* and *Arhopala* caterpillars might represent one of the first examples of such an arms race. As the main anti-herbivore defence of *Macaranga* trees via their ant partners has already been overcome by *Arhopala* caterpillars [24,31], the isolated occurrence of hooked trichomes in *M. trachyphylla* suggests that they evolved as a new defence to cope with heavy *Arhopala* herbivory. In turn, the ability of *A. amphimuta* caterpillars to walk over the hooked trichomes of *M. trachyphylla* without sustaining any injuries could represent an adaptation in response to this new defence. However, this arms race scenario is still speculative, as the detailed mechanisms underlying the resistance of *A. amphimuta* are still unknown, and it is equally possible that the relevant traits evolved as pre-adaptations for some other functions.

*A. amphimuta* caterpillars occur not only on *M. trachyphylla* but also on several other *Macaranga* host plants that are free of hooked trichomes. Since *M. trachyphylla* is endemic to Borneo, populations of *A. amphimuta* living on *M. bancana* and *M. hullettii* on the Malay peninsula have never encountered hooked trichomes. Future research on these populations could shed light on whether the resistance of *A. amphimuta* to hooked trichomes is indeed a counter-adaptation or a pre-adaptation that has allowed this species to survive on *M. trachyphylla*.

## 4. Methods

### (a) Field site

*Arhopala* caterpillars associated with *Macaranga* trees were studied in the Andulau Forest Reserve and the Ulu Temburong National Park in Brunei from May to June 2023 and from July to September 2024.

Each of the four *Arhopala* species is associated with a different set of *Macaranga* host plants (table 1). *Arhopala major* caterpillars were collected from *M. gigantea* trees (*n* = 25), *A. amphimuta* caterpillars from *M. bancana* (*n* = 17), *M. trachyphylla* (*n* = 5), *M. hullettii* (*n* = 4) and *M. aëtheadenia* (*n* = 1; to our knowledge, this is the first report of *M. aëtheadenia* as host plant of *A. amphimuta*). *Arhopala dajagaka* was collected from *M. rufescens* (*n* = 1), and *A. zylda* from *M. beccariana* (*n* = 3).

### (b) Trichome morphology

The morphology of hooked trichomes from leaves, stems and petioles of *M. trachyphylla* was studied using light and scanning electron microscopy. The density and height of trichomes on stems, petioles and leaves were measured on five samples each (from five different trees of 1−2 m height) using a Leica MZ16 stereomicroscope or a Leica DMR HC microscope (Leica Microsystems, Wetzlar, Germany) and photographed with a Nikon D750 DSLR camera (Nikon Corporation, Tokyo, Japan) mounted on it. Extended focus images were obtained by merging Z-stacks of images (20 µm interval) in Adobe Photoshop (Adobe Inc., CA, USA). The number of trichomes was counted within a selected area of 3 × 3 mm on the stem, petiole and the upper and lower leaf surfaces. The height of the trichomes (maximum extension perpendicular to the leaf surface) was measured in side view from cross sections of young leaves, stems or petioles (one cross section per plant and plant part, *n* = 5 plants). For electron microscopy, leaf, petiole and stem samples were first fixed overnight in formalin-alcoholic solution (4% v/v formaldehyde, 70% v/v ethanol), followed by gradual dehydration in an ethanol series (70%, 90% and absolute ethanol). The samples were then dried in a Quorum E3100 critical point dryer and sputter-coated with 20 nm of platinum (Quorum K575X; Quorum Technologies Ltd, East Grinstead, UK) before viewing them under the scanning electron microscope (Tescan MIRA3 FEG; TESCAN UK Ltd., Cambridge, UK) at 5 kV.

### (c) Effect of hooked trichomes on *Arhopala* caterpillars

Observations of caterpillars placed on leaves, petioles and stems of *M. trachyphylla* were made in Brunei under a Leica EZ4 stereomicroscope (Leica Microsystems, Wetzlar, Germany) and recorded with the camera of a OnePlus 11R mobile phone (OnePlus Technology Co., Ltd, China) attached to the microscope by an adapter (Celestron 81035). Twenty-five *A*. *major* caterpillars were studied (*n* = 10 first to second instar, *n* = 10 third to fourth instar and *n* = 5 fifth instar), as well as 27 *A*. *amphimuta* caterpillars (*n* = 10 first to second instar, *n* = 10 third to fourth instar and *n* = 7 fifth instar), three instar *A. zylda* (*n* = 1 third, *n* = 1 fourth instar and *n* = 1 fifth instar) and one first to second instar *A. dajagaka*. Each caterpillar was carefully placed on the surface using soft tweezers and then observed and video-recorded for 10 min under the stereomicroscope. One third to fourth instar caterpillar of *A. major* was freeze-dried after interacting with hooked trichomes on *M. trachyphylla* and viewed under the scanning electron microscope (see above) to visualize details of injuries.

### (d) Ant behaviour towards *Arhopala* caterpillars

Previous work showed that *A. major* caterpillars are able to feed on *M. trachyphylla* leaves under ant-free conditions in the laboratory, where they reached pupation at a rate even higher than on leaves of their natural host *M. gigantea* [30]. We therefore tested whether *A. major* caterpillars are accepted by the *Crematogaster* plant-ants living on *M. trachyphylla*. The ants’ behavioural responses were studied by placing *A. major* caterpillars on ant-inhabited *M. trachyphylla* plants [31]. Second, third or fourth instar *A. major* caterpillars were collected from *M. gigantea* in the field and kept on young ant-free leaves of their host plant in a plastic container in the laboratory for up to 2 days before the introduction. For the experiments, young *M. trachyphylla* trees of 1.5−2.0 m height with low herbivory damage were chosen, and one caterpillar was placed on the abaxial surface of the youngest or second-youngest visible leaf of the tree. The ants’ responses to the introduced larva were recorded for 10 min after the larva was approached for the first time by a worker of the resident ant colony. The reactions of ants that approached the caterpillar were divided into the following three categories (only one interaction was scored per ant visiting the leaf): (i) ignoring: ants did not attack or touch the caterpillar; (ii) attending: ants did not bite the caterpillar and touched them near the myrmecophilous glands on the posterior-dorsal side, sometimes drinking nectar; (iii) attacking: ants bit the caterpillar. Each caterpillar and each ant colony (tree) were only used once. At the end of the trials, the caterpillar, if not already killed by the ants, was placed on the first internode of the stem, and the caterpillar’s movements and ant behaviour were observed qualitatively for a further 10 min.

## Data Availability

All data are fully presented in the main text and supplementary material [34].
